# Tumor size as measured at initial X-ray examination, not length of bile duct stricture, predicts survival in patients with unresectable pancreatic cancer

**DOI:** 10.1186/1471-2407-12-429

**Published:** 2012-09-25

**Authors:** Henrik Forssell, Katrin Pröh, Michael Wester, Hans Krona

**Affiliations:** 1 Dept of Surgery, Blekinge Hospital, 371 85, Karlskrona, Sweden; 2 Blekinge Centre of Competence, Blekinge, 371 81, Karlskrona, Sweden; 3 Dept of Radiology, Blekinge Hospital, 371 85, Karlskrona, Sweden; 4 Blekinge Institute of Technology, School of Health Science, 371 79, Karlskrona, Sweden

**Keywords:** Pancreatic cancer, Pancreatic neoplasm, Unresectable, Tumor size, Biliary stricture, Palliative, Survival, Prediction of survival

## Abstract

**Background:**

The survival of unresectable pancreatic cancer patients is extremely poor. The aim of this study was to examine if tumor size could predict survival length in order to optimize patient care.

**Methods:**

A retrospective observational study was performed on 185 consecutive patients with unresectable pancreatic cancer (ICD10: C250-2 and C258) who were diagnosed from 2003 to May 2010. The patients' initial radiographs at presentation of symptoms were reviewed by the same radiologist, and tumor extent was determined.

**Results:**

The largest tumor diameter of the primary tumor was measured in 132 patients, 22 by an ultrasound and the other patients by a CT scan. In 53 patients, the tumor size could not be delimited and measured. Seventy-five patients (41%) had liver metastases at presentation of symptoms. Median survival for the entire patient group was only 119 days. The median diameter of the patient’s largest tumor was 4.35 cm, while the sample groups ranged from 1.2 to 14 cm. Patients were divided into two groups: those with a largest tumor diameter of ≤ 4.3 cm (66 patients) and those with a largest tumor diameter of > 4.3 cm (66 patients). Median survival for these groups was 149 and 94 days (p = 0.019), respectively. Cox regression showed a hazard ratio for tumor size of 1.48 (95% CI 1.02, 2.07) (p = 0.038), adjusted for the gemcitabine treatment which had been given to 49 patients and the presence of liver metastasis. In 88 patients, stricture length could be measured at ERCP. When comparing stricture lengths of ≤ 2 cm and > 2 cm, no difference in survival time was noted within a Kaplan-Meier analysis.

**Conclusion:**

The size of the maximum tumor diameter of the primary tumor during the initial X-ray examination of patients with pancreatic cancer may predict survival time for those patients who had no surgical resection. Stricture length at ERCP gave no information on survival.

## Background

Ductal pancreatic adenocarcinoma is one of the most highly fatal cancers and is the fourth or fifth leading cause of cancer death in the Western world. Five-year survival is less than 5%, and in advanced pancreatic cancer, five-year survival is zero [1,2]. The high mortality rate is due to the high incidence of metastatic disease at diagnosis. Thus the prognosis of patients with unresectable pancreatic carcinoma is extremely poor. The disease is difficult to treat because clinical presentation is often late, and most of the patients have an advanced tumor that is not resectable. Obstructive jaundice is the most common symptom requiring palliation and presents in 70% of patients, while duodenal or gastric outlet obstruction presents in 10-25% [3]. Each year nearly 900 new pancreatic tumors are diagnosed in Sweden. Less than 15% of these patients were treated by curative surgical resection of the tumor area. The majority of patients with locally advanced or metastatic tumor were thus given palliative treatment. Although new and better diagnostic methods have been developed, it is difficult to identify, evaluate and determine the size and spread of pancreatic cancer. Identification of biomarkers that accurately predict disease survival and recurrence or would provide clues for an individual risk assessment would be of great help but are not yet feasible today in the ordinary clinical setting [4], and methods such as radiographic investigations must be used to inform decisions regarding treatment choices and to predict survival length. Furthermore, it is not possible to determine an exact tumor node metastasis (TNM) classification status from an unresectable pancreatic neoplasm as it requires a pathological specimen, which can only be obtained from an operation. 

The purpose of this study was to examine whether tumor size or length of bile duct stricture at initial radiographic imaging may predict survival length in patients with inoperable pancreatic cancer.

## Methods

The study included consecutive patients diagnosed with ductal pancreatic cancer during the period January 2003 to May 2010 and recruited from a single medical centre. On starting the study all patients were deceased. Only patients with a diagnosis of locally advanced cancer in the caput, corpus and cauda of the pancreas, C250–2, or carcinoma growth outside the pancreas into adjacent organs, C258, according to ICD10 (International Classification of Diseases) were enrolled. All patients with jaundice received biliary plastic or metal stents. Patients who had surgery with curative purposes (i.e. Whipple procedure) were excluded in this survey. Initial X-rays following admittance to the hospital were reviewed by the same radiologist, and tumor size was measured again. Tumor size was determined in several ways. Some 22 patients underwent only ultrasonography, and specified size values from the statement were used in these cases. Most patients (161) were examined with an ordinary contrast enhanced multislice computed tomography (CT, slice thickness 2 mm), while only two patients were examined with magnetic resonance imaging (MRI). The surveys were reviewed and three size measurements were determined: width, length and depth of the tumor size. In 53 cases (29%), the tumor was too diffuse to be measurable. To simplify the calculations in this study, the maximum tumor diameter measured was that of the primary tumor. The median maximum tumor diameter was 4.35, and therefore the patient group was divided into two groups: 66 patients with ≤ 4.3 cm maximum tumor diameter and 66 patients with > 4.3 cm maximum tumor diameter. Stricture length affecting the bile duct was reexamined after the initial ERCP-examination in 66 patients. X-ray images were calibrated based on the diameter of the endoscope (11.7 mm). Twenty patients received percutaneous transhepatic cholangiography (PTC). These X-rays could not be calibrated afterwards, but the reported dimensions were used. Two patients underwent an initial magnetic resonance cholangiopancreatography (MRCP) and the reexamined stricture length was used. Stricture lengths affecting the bile duct were divided by frequency determination in two equal groups: ≤ 2 cm (44 patients) and > 2 cm (44 patients) since stricture length’s mean and median was 2 cm.

### Ethics

The study was approved by the Regional Ethical Review Board in Lund, Sweden (EPN Dnr 2012/92).

### Statistical analysis

The statistical analysis was performed using Stata version 12.1 (StataCorp LP, College Station, Texas, USA). Continuous variables are expressed as mean values with standard deviation (SD) and were compared by two sample Student’s *t*-tests. The comparison among groups for categorical variables was performed with Pearson chi2 tests. Overall survival estimates were calculated by the Kaplan-Meier method, and the difference between groups was assessed by the log rank test. Survival curves were truncated at 24 months since the number of patients at risk after that time was considered to be very small. Independent factors for overall survival were assessed with a Cox proportional-hazards regression analysis. P < 0.050 was considered statistically significant.

## Results

**Figure 1:**
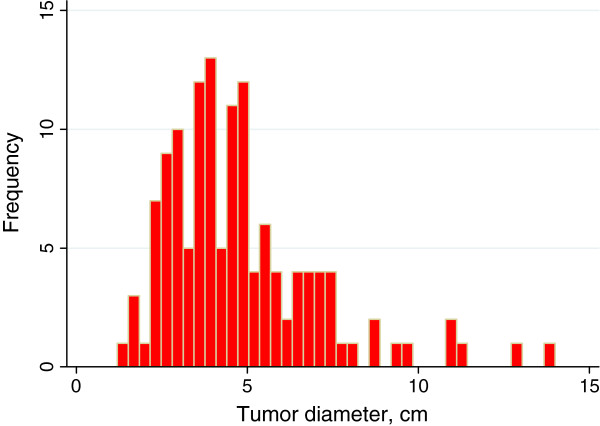
Distribution of the primary tumor's greatest diameter in 132 patients.

**Figure 2:**
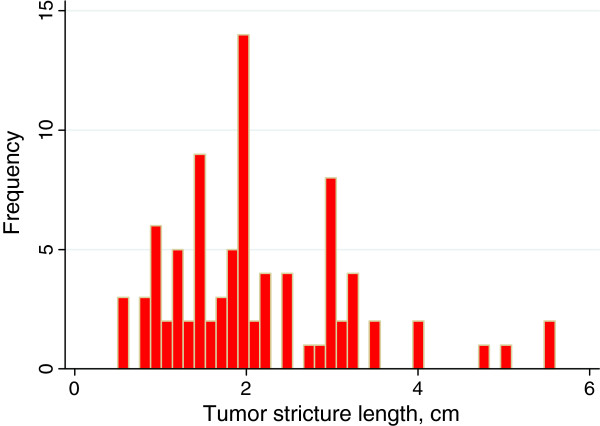
Distribution of bile duct stricture length in 88 patients.

**Figure 3:**
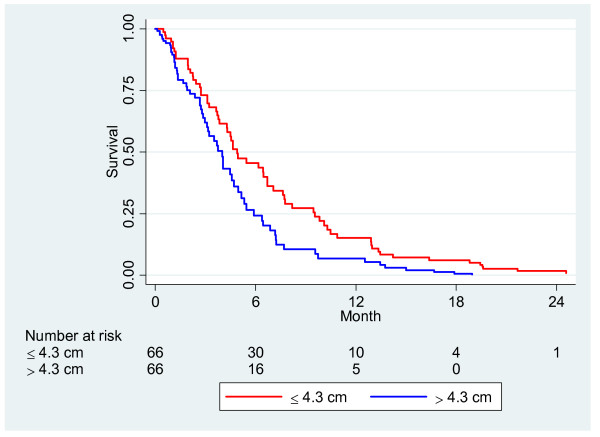
Survival analysis with tumor size in two groups: ≤ 4.3 cm, > 4.3 cm adjusted for presence of liver metastasis and treatment with gemcitabine, N = 132 (p = 0.038).

**Figure 4:**
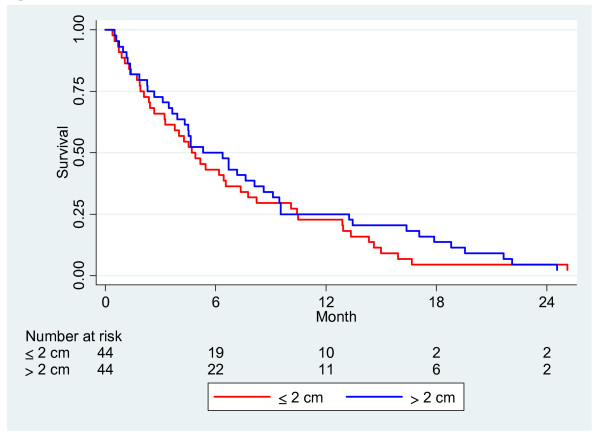
Survival analysis in patients with two groups of stricture length affecting the bile duct, N = 88 (p = 0.49).

A total of 185 patients were included in the study. The median age was 73.7 years old with a range of 43–92 years. Of these, 105 were women with a median age of 74.4 years old (43–92 years old) and 80 men with a median age of 72.7 years old (54–91 years old) (p = 0.30 for sex and age groups). Liver metastases were found in 75 patients (41%) at the initial radiological investigation. The largest tumor diameter was determined in 132 patients but could not be measured in the other 53 patients. Figure 1 shows the distribution of tumor size. The mean tumor diameter was 4.8 (SD 2.3) cm and median tumor size 4.35 cm with an interquartile range (IQR) of 2.5. Stricture of the bile duct due to cancer growth could be determined in 88 patients and the distribution is displayed in Figure 2. Mean stricture length was 2.1 cm (SD 1.1) and median stricture length was 2.0 cm (IQR 1.5). Most of the tumors were located in the caput region of the pancreas, Table 1. Patients who received gemcitabine were divided into two groups classified by if at least one cycle of gemcitabine were given or not. A cycle of gemcitabine consisted of three doses once a week for 3 weeks followed by a 1-week break in treatment. For the entire patient group, the median survival time was 119 days, and the survival of different tumor size groups is displayed in Table 2. The relationship between tumor size and survival is shown in Figure 3 where the survival curves are adjusted for gemcitabine treatment and occurrence of liver metastasis, and a significant difference was found to be consistent throughout the entire patient group. (p = 0.038). Bile duct stricture could be analyzed in 88 patients, and the median length was 2.0 cm. Survival time was not longer in patients with stricture length of less than 2 cm compared to those with a larger stricture length, Figure 4. Stricture length had no predictive value on survival rate analyses, according to the Cox regression (p = 0.730). There was also no correlation between stricture length and tumor size. A Cox regression was performed on tumor size, gemcitabine treatment and liver metastasis (Table 3). Only 49 of 132 patients with measurable tumor received gemcitabine, the median cycle number was 4 (range 1 to 16 cycles).

**Table 1:** Baseline characteristics of patients with primary pancreatic neoplasm

**All patients,**	**185**
Female/ male	105/80
Mean age (SD), year	73.7 (10.7)
Visible tumor	132
Not possible to measure	53
Liver metastasis (%)	75 (41)
Patients with visible tumor	132
Mean age (SD), year	72.6 (11.0)
Female/male	75/57
Liver metastasis (%)	60 (45)
Tumor diameter ≤ 4.3 cm	66
Tumor diameter > 4.3 cm	66
Gemcitabine treatment (%)	49 (37)

**Table 2:** Median survival time in days according to tumor size and liver metastasis

**Tumor size**	**N**	**All patients**	**No liver metastasis**	**Liver metastasis**
All studied patients	185	119	179	83
Diameter ≤ 4.3 cm	66	149	204	111
Diameter > 4.3 cm	66	94	157	58

**Table 3:** Multivariable Cox regression in 132 patients with measurable tumor The model had a chi2 value of 55.5 with 3 degrees of freedom

	**Hazard ratio**	**95% Conf. Interval**		**P**
Tumor size: ≤ 4.3 cm, > 4.3 cm	1.45	1.02	2.07	0.038
Gemcitabine (no, yes)	0.33	0.22	0.49	<0.001
Liver metastasis (no, yes)				

## Discussion

In the current study, the median survival time for all 185 patients with unresectable ductal pancreatic adenocarcinoma was only 119 days, which is about the same survival length reported by others [5,6]. The study showed that tumor size of the primary tumor as measured at the patient’s initial X-ray examination may predict survival length, especially if the presence of liver metastasis and gemcitabine therapy was taken into consideration and adjusted for in the Cox regression. Stricture length measured at ERCP was not a factor that could predict survival duration. Stricture length was divided into two groups (less than or equal to and greater than 2 cm) in 88 patients, and a survival analysis using the log rank test showed no significant difference at all, even when survival analysis was adjusted for gemcitabine in a Cox regression. Therefore, stricture length is an irrelevant prognosis factor for pancreatic cancer, and this has not been described previously. It is likely that the tumor grows asymmetrically and stricture length is a misleading indicator of the actual tumor size. If liver metastasis exists, patients have a significantly shorter survival compared with patients without primary liver metastasis. The difference was about 3 months. Even when the pancreatic tumor was less than 4.3 cm, there was a significant difference in survival time regarding liver metastasis or not. Tumor biology is certainly important and may partially explain the difference found between the groups. The findings are in accord with a recent German study where the only independent prognostic factor for shorter survival was distant metastasis [7]. A multivariable approach was used to account for the functional form of the relationship between continuous prognostic variable factors and survival in advanced pancreatic cancer [5]. The study investigated multiple clinical, histological, biochemical and demographic variables in the form of both binary and continuous measurements. The model confirmed five prognostic factors, namely albumin, CA19-9, alkaline phosphatase, lactate dehydrogenase and metastases; and also identified three additional possible prognostic factors as: leukocytes, aspartate aminotransferase and blood urea nitrogen. Reported hazard ratios were between 2.08 and 1.50. Tumor size was not included in the report, however, which our study identified as an important variable for predicting survival. Only early or late tumor stages were used, and no significant difference in survival time was found [5]. Prognostic factors for survival have been described for patients who have undergone a surgical resection to treat their pancreatic cancer. But in the group of patients with advanced adenocarcinoma, detailed data on prognosis regarding tumor size and stricture length is lacking. Some studies have focused on the value of CT criteria in predicting survival rates for patients with potentially resectable pancreatic head carcinoma [8,9]. One of these studies included a small number of unresectable patients and showed a poor 4 month survival rate for these patients, but the only significant factors were whether the superior mesenteric artery was encased by the neoplasm or not and whether the tumor size was more than 3 cm [8]. The results from another study suggested that baseline performance status activity and the occurrence of distant metastasis were the only variables that could be independently associated with predicting survival in patients with unresectable pancreatic carcinoma [10]. In this study, the median survival was only 94 days compared to the 119 days found in the present study. This indicates that our study included patients with an advanced disease status and that survival was better predicted by using tumor size at the initial X-ray examination than relying on baseline performance status activity. Imaging studies play a crucial role in the diagnosis and management of patients with ductal pancreatic adenocarcinoma. Multislice CT is the most widely available and best-validated modality for imaging patients with pancreatic neoplasm. The sensitivity of CT for diagnosis of pancreatic adenocarcinoma, ranging 50–97%, and its positive predictive value for predicting unresectability, ranging 80 - 100%, are high but decreases for pancreatic lesions smaller than 1.5 to 2 cm [11]. In our study, however, 29% of patients with unresectable cancer had tumors that could not be defined or measured. Newer CT machines with better image quality should make it easier to find small tumors. In the future, better CT or MRI computer protocols may improve the detection of pancreatic tumors but at present, semiautomatic computer programs are expected to render a measurement difference of > 10% between radiologists [12]. Tumor volume as measured by different radiological methods and calculated as a square or bullet may lead to overestimations of tumor volume. It would be better to use programs that recognize the tumor in the pancreas and automatically calculate the volume. Parlak et al. assessed whether gross tumor volume determined by fusion of contrast-enhanced computerized tomography (CT) and 18 F-fluoro-deoxy-D-glucose positron emission tomography-CT (FDG-PET-CT) based radiotherapy could predict overall survival in 30 patients with advanced pancreatic neoplasm and found that a volume of 91 cm^3^ corresponding to a tumor diameter of 5,58 cm predicted well survival [13]. They found 16.3 vs. 9.5 months of survival difference. However survival rates were determined during radiotherapy and concurrent continuously infused 5-FU followed by 4 to 6 courses of maintenance gemcitabine and it is unclear if they adjusted for the treatment.

Endoscopic ultrasound (EUS) is considered superior to CT and MRI, especially in detecting small tumors and lymph node metastasis. EUS can also be combined with fine needle aspiration (FNA) and has a sensitivity of 85–95% and a specificity of 97–100% [14]. Newer modalities in conjunction with EUS are contrast-enhanced EUS and tridimensional EUS, but all are operator dependent. It is reasonable to state that the tumor size has a significant impact on survival duration. This study used ordinary diagnostic tools but although clearly illustrates that survival rates are higher for patients with small tumors. Cox regression confirmed this finding even when the data was adjusted for gemcitabine doses and the presence of liver metastasis. Consequently, determining the largest tumor diameter at the patient’s initial X-ray examination and presence of liver metastasis can identify patients with a very short survival time and those who are likely to survive longer in order to allow for optimal individualized patient care.

## Conclusion

Determining the maximum tumor diameter of the primary tumor at the initial X-ray examination, but not length of stricture at ERCP can predict survival time for patients with unresectable pancreatic cancer.

## Competing interests

No competing interests exist.

## Authors’ contributions

All authors substantially contributed to the current manuscript as listed below. HF drafted the manuscript, is the principal investigator of the study and designed this study. KP reviewed and measured tumor size of all X-rays. MW, KP, HK revised the manuscript. All authors read and approved the final manuscript.

## Pre-publication history

The pre-publication history for this paper can be accessed here:

http://www.biomedcentral.com/1471-2407/12/429/prepub
